# Supplementary material for: MetaRibo-Seq measures translation in microbiomes
Source: Nat Commun. 2020 Jun 29;11:3268. doi: 10.1038/s41467-020-17081-z (PMC7324362; doi:10.1038/s41467-020-17081-z)
Supplement: Supplementary file 10 — Supplementary Data 7 [file 41467_2020_17081_MOESM10_ESM.zip › File2/Confidence_High_Taxonomy/162663_out.krona.html]

Javascript must be enabled to view this page.

members
magnitude
magnitudeUnassigned
count
unassigned
taxon
rank

162663\_out

2

2
superkingdom
2

2
phylum
201174

class
2
1760

order
2
2037

2049
family
2

genus
2
1654

1655

SRS019176\_contig\_number\_17724SRS077075\_contig\_number\_contig-100\_34424.34424
species
2
